# Enhancing Caudal Analgesia in Pediatrics: A Comparison of Ropivacaine With and Without Clonidine for Infra-Umbilical Surgeries

**DOI:** 10.7759/cureus.68979

**Published:** 2024-09-09

**Authors:** Sonal Khatavkar, Veda Sumi Durgumpudi

**Affiliations:** 1 Anesthesiology, Dr. D. Y. Patil Medical College, Hospital & Research Centre, Dr. D. Y. Patil Vidyapeeth (Deemed to be University), Pune, IND

**Keywords:** caudal block, clonidine, pediatric anesthesia, postoperative analgesia, ropivacaine

## Abstract

Introduction

Regional anesthesia, particularly caudal blocks, is increasingly used in pediatric surgeries for effective post-operative pain management. However, the duration of analgesia with agents such as ropivacaine alone can be limited. This study investigates the effects of adding clonidine to ropivacaine in caudal blocks for pediatric patients undergoing infra-umbilical surgeries, with the purpose of enhancing the duration and quality of analgesia.

Methods

We conducted a randomized, double-blind study including 50 children aged one to eight years, divided equally into two groups: group R received 0.2% ropivacaine and group RC received 0.2% ropivacaine with 2 mcg/kg clonidine. Intraoperative and post-operative monitoring included heart rate, blood pressure, and respiratory parameters. Analgesia duration, sedation scores, and the need for rescue analgesia were assessed.

Results

Group RC exhibited significantly longer analgesia duration (18.4 ± 2.31 hours) compared to group R (10.56 ± 2.27 hours, P < 0.0001). Fewer patients in group RC required a second dose of rescue analgesia (4% vs. 32%, P = 0.023), with no significant differences in sedation scores or adverse effects between the groups.

Conclusion

Adding clonidine to ropivacaine in caudal blocks significantly prolongs analgesia and reduces the need for additional post-operative pain management in pediatric surgeries without increasing the risk of side effects. This study supports the use of clonidine as an effective adjuvant in pediatric pain management.

## Introduction

In recent decades, regional anesthesia in children has gained widespread acceptance, largely because of its benefits such as reduced post-operative pain, decreased opioid use, lower pain scores, fewer respiratory issues, and reduced nausea. In pediatric patients, potent post-operative pain management is essential yet challenging. The caudal block is a valuable adjuvant to general anesthesia for infra-umbilical surgeries, offering effective pain relief with agents such as bupivacaine and ropivacaine. However, these agents alone provide a shorter block duration, necessitating additional post-operative analgesia [1]. The gradual onset and offset make it ideal for outpatient procedures, reducing the need for intravenous pain medications and supporting early discharge.

Ropivacaine is preferred over bupivacaine for its lower toxicity and reduced motor blockade [2]. Higher concentrations of ropivacaine provide long-lasting analgesia but may cause toxicity in children. Adjuvants such as opioids or ketamine can extend the block duration. Clonidine, once an antihypertensive, is now used to enhance analgesia by inhibiting nociceptive neurotransmitters without causing significant respiratory depression [3].

Our study aims to evaluate the perioperative analgesic efficacy of 0.2% ropivacaine, with and without clonidine, in caudal blocks for children aged one to eight years undergoing infra-umbilical procedures.

## Materials and methods

Following the authorization from the Institutional Ethics Committee of Department of Anesthesiology, Dr. D. Y Patil Medical College, Hospital & Research Centre, Pimpri, Pune, Maharashtra, India, and the Clinical Trials Registry-India (CTRI/2023/09/058101), we conducted a six-month study, including 50 American Society of Anesthesiologists (ASA) grading I and II children, aged one to eight years, scheduled for elective below-umbilical surgeries. Informed consent was acquired from parents. Exclusion criteria were local infection at the caudal site, coagulation disorders, prior neurological or spinal conditions, congenital spine deformities, ASA III or higher, and allergies to the study medications. The study was conducted in a computer-generated random, double-blind manner. The sample size for this study was calculated using WinPepi software version 11.7, based on the duration of analgesia, with the mean and standard deviation derived from the study by Bajwa et al., which involved 60 patients [4]. We did not consider an attrition rate in our study, as the patients were admitted for at least post-operative day 1, and the study was limited to 24 hours of observation.

A senior anesthesiologist not involved in the study prepared the drug, following written protocols to load sterile syringes with the same volume of the placebo (normal saline) or drug. Another anesthesiologist, unaware of the syringe contents, administered the drug and conducted the intraoperative and post-operative monitoring.

Standard ASA monitors (electrocardiography, non-invasive blood pressure (NIBP), oxygen saturation [SpO_2_], end-tidal CO_2_ [ETCO_2_]) were attached to the patient, and premedication midazolam 0.02 mg/kg intravenously (IV) and glycopyrrolate 0.004 IV were given 10 minutes before surgery in the operation room (OR). All patients received fentanyl 1 mcg/kg IV, followed by induction with propofol 2-3 mg/kg IV, and were then mask-ventilated before inserting an i-gel™ laryngeal mask airway (LMA) (Intersurgical Ltd., Wokingham, Berkshire, UK) of appropriate size. Anesthesia was continued on 50% FiO_2_ and sevoflurane dial at 1.5%-2%, maintaining patients on spontaneous ventilation.

Patients were positioned in the left lateral Sims position, vitals were reassessed, and adequate spontaneous breathing was ensured. Under all aseptic precautions, using thumb palpating from the coccyx to the sacrum, the sacral hiatus was located. A 22G hypodermic needle at a 60°-70° angle bevel facing anteriorly was inserted until the sacrococcygeal membrane was pierced, giving a feeling of "pop." The needle was then adjusted to a 20° angle and advanced 2-3 mm. Swoosh test, which involves injecting a small amount of saline to listen for a characteristic “whoosh” sound indicating correct placement in the epidural space, and lack of resistance with a 1-mL normal saline injection were used to confirm the needle position [5]. To exclude dural or vessel puncture, negative aspiration was performed before drug injection:

Group R: 25 patients received 0.2% ropivacaine (1 mL/kg) in a caudal block with 0.5 mL of normal saline.

Group RC: 25 patients received 0.2% ropivacaine (1 mL/kg) with clonidine (2 mcg/kg).

Following the injection, the child was positioned supine, with no further analgesia administered intraoperatively. Anesthesia was maintained using oxygen, air, and sevoflurane (0.5-2%) via an i-gel LMA, with the patient on spontaneous ventilation throughout the surgery. Vitals including heart rate (HR), blood pressure, SpO_2_, and ETCO_2_ were monitored. Ringer lactate with 1% dextrose solution was used for IV fluids and given according to the child's body weight and fasting status.

Recovery

Upon discontinuing anesthetic agents after closure, the i-gel LMA was removed, and the child was observed in OR for 5 minutes on room air. Once stable and awake, the child was positioned in semi-prone and monitored for Face, Legs, Activity, Cry, Consolability (FLACC) scale score [6], Ramsay sedation score, and vital parameters (SpO_2_, respiratory rate, NIBP, HR) at 15, 30, 60, 90, and 120 minutes in the post-anesthesia care unit (PACU). Subsequently, monitoring continued every 4 hours in the ward for 24 hours. Post-operative, FLACC scores for post-operative analgesia and sedation scores were assessed. If FLACC scores exceeded 4, rescue analgesia with 15 mg/kg IV paracetamol was administered. The patients were also observed for adverse effects such as vomiting, urinary retention, and respiratory depression.

## Results

**Figure 1:**
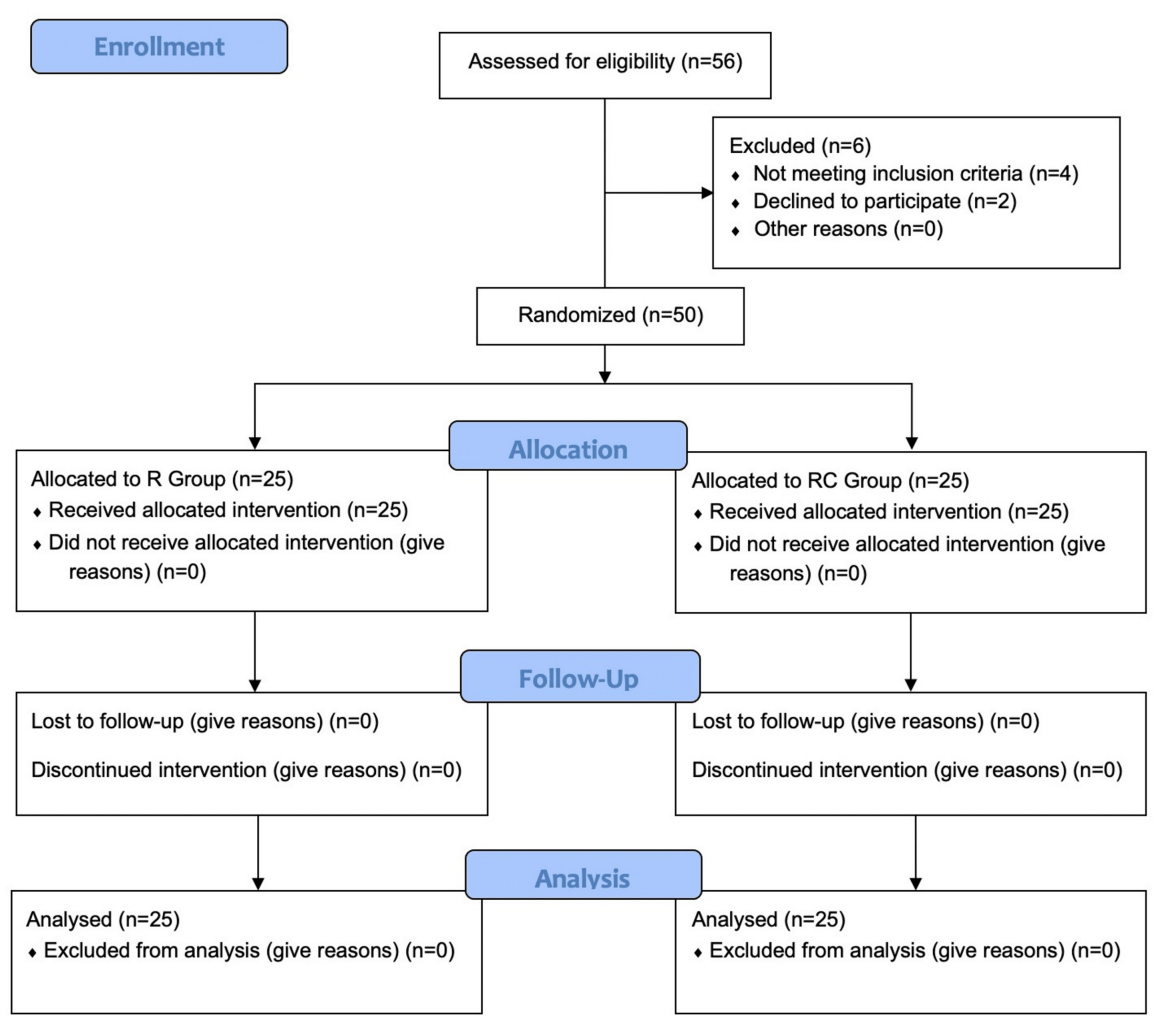
CONSORT flow diagram showing participants’ progress through the phases of the randomized controlled trial

This randomized controlled trial included 50 participants, who were equally allocated into two groups, group R and group RC, each consisting of 25 participants. Initially, 56 participants were assessed for eligibility, but six were excluded due to not meeting the inclusion criteria or declining to participate (Figure 1). Both groups completed the study without any losses to follow-up, and all participants were included in the final analysis.

Table 1 compares the demographic parameters between the two groups, which were similar in terms of age, weight, and gender distribution, with no statistically significant differences (P > 0.05). Herniotomy was the most common surgery in both groups (56% in group R and 52% in group RC). Circumcision was more common in group RC (32% as compared to 16% in group R), while hypospadias repair and orchidopexy were performed in both groups at similar rates. The overall comparison indicates no significant differences in the distribution of these parameters between the groups.

**Table 1:** Comparison of demographic parameters (N = 50) †Independent t-test, *Fisher's exact test

Parameters	Group R (n=25)	Group RC (n=25)	P-value
Age (years), mean ± SD	4 ± 1.35	3.48 ± 1.41	0.187^†^
Weight (kilograms), mean ± SD	11.88 ± 2.91	11.52 ± 2.86	0.661^†^
Gender			
Male	24 (96%)	22 (88%)	0.609^*^
Female	1 (4%)	3 (12%)	
Type of surgeries			
Circumcision	4 (16%)	8 (32%)	0.555^*^
Herniotomy	14 (56%)	13 (52%)	
Hypospadias repair	3 (12%)	2 (8%)	
Orchidopexy	4 (16%)	2 (8%)	

**Figure 2:**
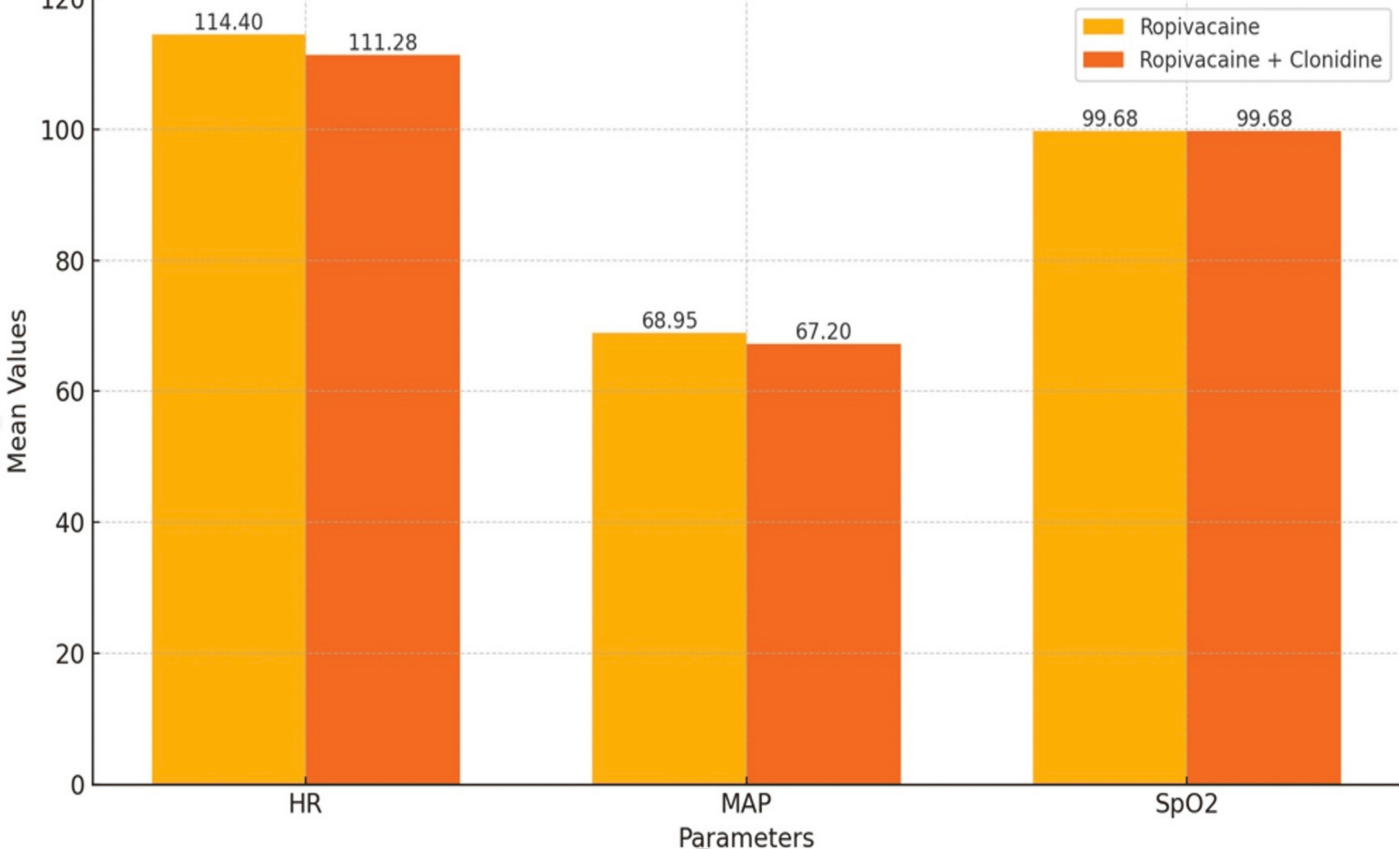
Comparison of vitals in both groups HR, heart rate; MAP, mean arterial pressure; SpO_2_, oxygen saturation

Figure 2 illustrates a comparative analysis of intraoperative mean HR, mean arterial pressure (MAP), and mean SpO_2_ that exhibited no statistically significant differences (P > 0.05). Additionally, the duration of surgery or the time required for extubation after anesthesia cessation in both groups did not differ significantly (P > 0.05). None of the patients experienced notable hypotension or bradycardia (drop of more than 20% from normal baseline). Throughout the procedure, SpO_2_ levels consistently remained >97% in both groups (P > 0.05).

**Figure 3:**
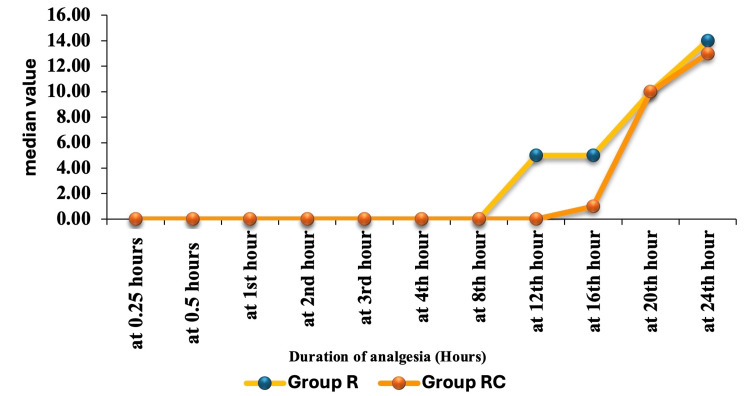
Correlation of duration of analgesia (hours) between group R and group RC.

Figure 3 shows that both group R and group RC had no pain (FLACC score of 0) until the 8th hour. After the 12th hour, pain scores in group R increased sharply, while group RC maintained lower scores until the 16th hour, indicating better and longer-lasting pain control in group RC.

As shown in Table 2, group RC had a significantly longer period of analgesia compared to group R, with a mean of 18.4 ± 2.31 hours versus 10.56 ± 2.27 hours (P < 0.0001). Most patients in group RC had analgesia lasting 16-20 hours, while group R's duration was predominantly 8-12 hours. The difference in analgesia duration between the groups is statistically significant (P < 0.0001).

**Table 2:** Correlation of duration of analgesia (hours) between group R and group RC. †Independent t-test, *Fisher's exact test SD, standard deviation

Duration of analgesia (hours)	Group R (n=25)	Group RC (n=25)	Total	P-value
8	10 (40%)	0 (0%)	10 (20%)	<0.0001^*^
12	14 (56%)	0 (0%)	14 (28%)	
16	1 (4%)	11 (44%)	12 (24%)	
20	0 (0%)	13 (52%)	13 (26%)	
24	0 (0%)	1 (4%)	1 (2%)	
Mean ± SD	10.56 ± 2.27	18.4 ± 2.31	14.48 ± 4.56	<0.0001^†^
Median (25th-75th percentile)	12(8-12)	20(16-20)	16(12-20)	
Range	8-16	16-24	8-24	

As shown in Table 3, sedation scores between group R and group RC were similar (P = 0.763). However, significantly fewer patients in group RC required a second dose of rescue analgesia compared to group R (4% vs. 32%, P = 0.023), indicating better pain management in group RC.

**Table 3:** Comparison of sedation scores and rescue analgesia requirements between group R and group RC †Independent t-test, *Fisher's exact test

Characteristics	Group R	Group RC	P-value
Mean sedation score	1.32 ±0.48	1.28 ±0.46	0.763^†^
Rescue analgesia second dose requirement	8 (32%)	1 (4%)	0.023^*^

Table 4 shows a comparison of side effect incidences between the two groups. In group RC, two patients experienced vomiting, compared to one patient in group R, a difference that was statistically insignificant (P > 0.05). Urinary retention was noted in both groups and was managed with bladder monitoring and, if necessary, catheterization, while vomiting was managed with antiemetics (Inj. ondansetron 0.1 mg/kg) and adequate hydration. No additional adverse side effects were observed in either group.

**Table 4:** Comparison of post-operative side effects between group R and group RC. *Fisher's exact test

Side effects	Group R (n=25)	Group RC (n=25)	Total	P-value
None	22 (88%)	21 (84%)	43 (86%)	1.00^*^
Urinary retention	2 (8%)	2 (8%)	4 (8%)	
Vomiting	1 (4%)	2 (8%)	3 (6%)	
Total	25 (100%)	25 (100%)	50 (100%)	

## Discussion

Clonidine has gained increasing attention in pediatric anesthesia as an adjuvant that enhances the effects of local anesthetics. By acting on alpha-2 adrenergic receptors, clonidine modulates nociceptive transmission and extends the duration of analgesia, making it a valuable agent for improving pain management in pediatric patients. Several studies have explored its effectiveness, demonstrating its ability to prolong post-operative analgesia when combined with local anesthetics such as ropivacaine, which, due to its reduced toxicity and lower risk of motor blockade, is already favored in pediatric populations. This study, consistent with existing literature, highlights the benefits of adding clonidine (2 mcg/kg) to 0.2% ropivacaine for caudal analgesia in children undergoing infra-umbilical surgeries.

The choice of ropivacaine over bupivacaine for pediatric caudal blocks is due to its lower risk of motor blockade and reduced systemic toxicity. However, rare cases of ropivacaine-induced convulsions and severe cardiac dysrhythmia have been reported, as highlighted by Ruetsch et al. [7]. Despite these concerns, our study observed no such adverse effects, likely due to the low concentration of ropivacaine used and the addition of clonidine, which has been shown to enhance safety profiles in regional anesthesia.

Clonidine’s utility as an analgesic adjuvant stems from its action on alpha-2 adrenergic receptors, where it suppresses nociceptive transmission in the spinal cord, extending the duration of local anesthetics such as ropivacaine. Manickam et al. demonstrated that clonidine significantly enhances the analgesic duration of ropivacaine in pediatric patients, which is consistent with our finding of prolonged analgesia in the clonidine group [8]. Similarly, meta-analyses by Pöpping et al. and Wang et al. support the efficacy of clonidine in peripheral nerve blocks, suggesting it significantly improves analgesic outcomes [9,10].

In pediatric anesthesia, caudal blocks are a preferred regional technique for infra-umbilical surgeries. Studies by Cook et al. and de Beer and Thomas have confirmed the role of clonidine in extending the duration of caudal analgesia without significant hemodynamic or respiratory complications, as observed in our study [11,12].

Furthermore, the combination of ropivacaine and clonidine results in a significant reduction in post-operative analgesic requirements. Shukla et al. and Laha et al. similarly reported that adding clonidine to caudal anesthesia reduced the need for additional analgesics post-operatively, which aligns with our findings [13,14]. This opioid-sparing effect is clinically important, as it minimizes the potential side effects associated with higher doses of post-operative opioids or non-opioid analgesics.

Our study also noted no significant sedation in patients who received clonidine, a finding supported by Hansen et al., who demonstrated that clonidine did not cause excessive sedation when used in pediatric caudal blocks [15]. While Ivani et al. reported dose-dependent post-operative sedation in children receiving clonidine with bupivacaine [16], our study, using a lower dose of clonidine (2 mcg/kg), showed no significant sedation between the groups, with all patients easily arousable.

One of the key advantages of ropivacaine is its reduced incidence of motor blockade compared to bupivacaine. Studies such as those by Zaric et al. and Ivani et al. have demonstrated that low concentrations of ropivacaine result in less post-operative motor block, which is a critical consideration in pediatric patients where early mobility is desirable [17,18]. Our study observed no motor deficits in either group, further confirming ropivacaine’s safety in this context

The study's methodology ensured a robust and unbiased comparison. The double-blind, randomized design, along with the preparation and administration of drugs by different anesthesiologists, minimized potential biases. Standardized premedication, induction, and maintenance protocols further strengthened the validity of our findings.

However, some limitations should be considered. The study was limited to the age group of one to eight years undergoing infra-umbilical surgeries, which may restrict the generalizability of the results to other age groups or surgical procedures. Additionally, the follow-up period was limited to 24 hours post-surgery, which may not capture late-onset side effects or prolonged analgesic effects.

## Conclusions

Our study demonstrated that adding 2 mcg/kg clonidine to 0.2% ropivacaine for caudal blocks in pediatric infra-umbilical surgeries significantly prolongs the duration of analgesia and reduces the need for additional post-operative pain management without increasing the incidence of significant side effects. The enhanced analgesic efficacy observed in group RC supports the use of clonidine as an effective adjuvant. Despite some limitations, the findings align with previous research, underscoring clonidine's role in improving pediatric pain management. The double-blind, randomized design ensured robust and unbiased results, highlighting the potential for clonidine to enhance the safety and effectiveness of caudal anesthesia in children.
